# Differential Glucose-Regulation of MicroRNAs in Pancreatic Islets of Non-Obese Type 2 Diabetes Model Goto-Kakizaki Rat

**DOI:** 10.1371/journal.pone.0018613

**Published:** 2011-04-07

**Authors:** Jonathan Lou S. Esguerra, Caroline Bolmeson, Corrado M. Cilio, Lena Eliasson

**Affiliations:** 1 Islet Cell Exocytosis, Department of Clinical Sciences-Malmö, Lund University, Malmö, Sweden; 2 Cellular Autoimmunity Unit, Department of Clinical Sciences-Malmö, Lund University, Malmö, Sweden; University of Bremen, Germany

## Abstract

**Background:**

The Goto-Kakizaki (GK) rat is a well-studied non-obese spontaneous type 2 diabetes (T2D) animal model characterized by impaired glucose-stimulated insulin secretion (GSIS) in the pancreatic beta cells. MicroRNAs (miRNAs) are short regulatory RNAs involved in many fundamental biological processes. We aim to identify miRNAs that are differentially-expressed in the pancreatic islets of the GK rats and investigate both their short- and long term glucose-dependence during glucose-stimulatory conditions.

**Methodology/Principal Findings:**

Global profiling of 348 miRNAs in the islets of GK rats and Wistar controls (females, 60 days, N = 6 for both sets) using locked nucleic acid (LNA)-based microarrays allowed for the clear separation of the two groups. Significant analysis of microarrays (SAM) identified 30 differentially-expressed miRNAs, 24 of which are predominantly upregulated in the GK rat islets. Monitoring of qPCR-validated miRNAs during GSIS experiments on isolated islets showed disparate expression trajectories between GK and controls indicating distinct short- and long-term glucose dependence. We specifically found expression of rno-miR-130a, rno-miR-132, rno-miR-212 and rno-miR-335 to be regulated by hyperglycaemia. The putative targets of upregulated miRNAs in the GK, filtered with glucose-regulated mRNAs, were found to be enriched for insulin-secretion genes known to be downregulated in T2D patients. Finally, the binding of rno-miR-335 to a fragment of the 3′UTR of one of known down-regulated exocytotic genes in GK islets, Stxbp1 was shown by luciferase assay.

**Conclusions/Significance:**

The perturbed miRNA network found in the GK rat islets is indicative of a system-wide impairment in the regulation of genes important for the normal functions of pancreatic islets, particularly in processes involving insulin secretion during glucose stimulatory conditions. Our findings suggest that the reduced insulin secretion observed in the GK rat may be partly due to upregulated miRNA expression leading to decreased production of key proteins of the insulin exocytotic machinery.

## Introduction

MicroRNAs (miRNAs) are ∼21-23 nucleotides (nt) long, non-coding RNAs which negatively regulate expression of genes via mRNA degradation, mRNA deadenylation and/or translational repression [1]. However, during the cell cycle arrest some miRNAs may mediate translational activation of specific transcripts [2]. A single miRNA may have multiple target genes and it is estimated that 20% of all mammalian genes may be direct targets for post-transcriptional silencing [3]. Thus one miRNA is capable of causing widespread changes in the proteins synthesis for thousands of genes [4]. MiRNAs have been shown to be involved in various aspects of fundamental cellular and physiological processes such as in cell differentiation, proliferation, apoptosis, morphogenesis, fat metabolism, hormone secretion, and long-term memory [5]. Hence deregulated miRNA expression has been implicated in many diseases, particularly in many different types of human cancer [6], [7], in cardiovascular and skeletal muscle diseases [8] and in neurodegenerative diseases such as Alzheimer's disease [9] and amyotrophic lateral sclerosis (ALS) [10].

The link between miRNA and diabetes began with the discovery of a highly-expressed pancreatic islet miRNA, miR-375. It was then shown to be involved in exocytosis via the targeting of the myotrophin gene in a still unclear mechanism [11]. Recently it was shown that miR-375 knockout mice display elevated plasma glucose levels due to a small reduction in beta cell mass with a concomitant increase in the number of alpha cells per islet as well as increased circulating glucagon [12]. Currently, a number of other miRNAs have been implicated in the proper functioning of the insulin-secreting beta cell, albeit mostly in cultured cell lines [13].

The Goto-Kakizaki (GK) rat, selectively bred from Wistar rats with high blood glucose levels, is a well-studied non-obese spontaneous type 2 diabetes animal model [14]. Its main associated diabetic phenotype regardless of colony source is impaired glucose-stimulated insulin secretion (GSIS) [15], [16]. Indeed genetic lesions in the GK rat point to defects in vital components of the canonical secretion coupling of the pancreatic islet beta cell. In particular, reduced expression of a number of exocytotic soluble *N*-ethylmaleimide-sensitive factor attachment protein receptor (SNARE) complex proteins has been shown in GK rat islets [17], [18], similar to those observed in type 2 diabetic humans [19].

The contribution of miRNAs to the development of diabetes in the GK rat has been largely unexplored. Insulin resistance in target tissues is one aspect of the GK diabetic phenotype and miRNA profiling studies on skeletal muscle, liver and adipose tissues of the diseased animal have been performed [20], [21]. However, there has been no systematic study on the GK pancreatic islet miRNAs. Here we investigated the miRNA expression profiles in the pancreatic islets of the GK rat compared to the Wistar controls and evaluated both the short term and long term glucose-dependence of differentially-expressed miRNAs.

## Results

### Phenotype of GK/MolTac versus aged-matched Wistar rats

**Figure 1:**
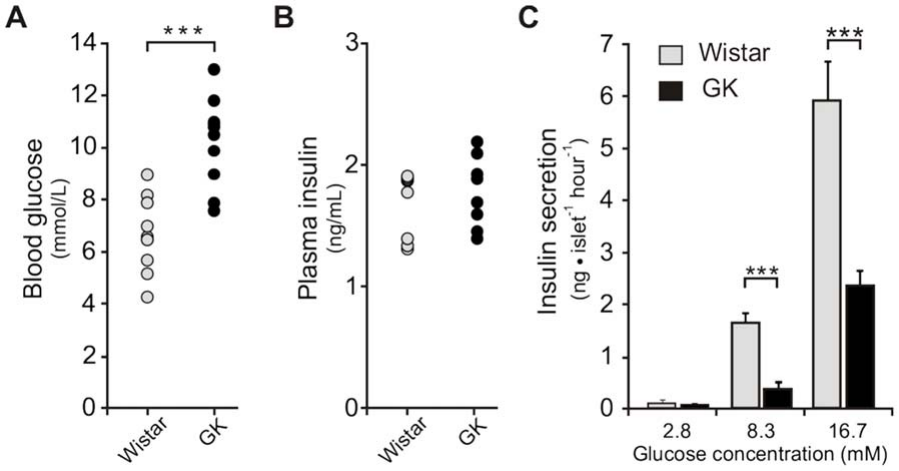
Phenotype of the GK rats at the time of islets collection. **A**. Non-fasting intra-cardial blood glucose levels are elevated in the GK rats compared to non-diabetic Wistar controls (*N* = 10 in both groups). **B**. Plasma insulin levels are at comparable levels between the two groups of animals (*N* = 8 in both groups). **C**. Insulin secretion is reduced in the isolated pancreatic islets of GK rat at 8.3 mM and 16.7 mM glucose (*N* = 3 independent RIA in quadruplicate per assay). Data are average ± SEM; (***) *P*<0.001 GK *vs* Wistar.

Consistent with previous studies on 8-week old Stockholm colony GK rats [22] as well as in GK/MolTac subline [23], we observed significantly elevated non-fasting blood glucose levels in our rats (10.2±0.5 mM, *N* = 10) compared to the age-matched Wistar controls (6.7±0.4 mM, *N = *10) (p = 7.5×10^−5^) (Fig. 1A). The plasma insulin levels displayed non-significant differences between the two groups of animals (Fig. 1B), though there is a tendency for the GK rats at this age to exhibit elevated circulating insulin consistent with hyperglycaemia and impending diabetic state as the animals get older [23].

Another well-known phenotype of GK rat is insulin resistance in target tissues. In the aforementioned study of Ostenson and co-workers, results of intra-peritoneal glucose tolerance test showed rapid increase in blood glucose to a maximum 20 mmol/L (*vs* 8 mmol/L in Wistar control). After 120 minutes, the blood glucose in the GK was still twice its fasting level clearly indicating an impaired insulin response [22].

A hallmark feature of diabetic GK rats regardless of sub-line is impaired GSIS whether in *in vivo* studies, perfused pancreas or in freshly isolated islets (reviewed in [16]). Indeed, in agreement with other studies on isolated GK islets, we observed reduced insulin response in islets stimulated in 8.3 mM and 16.7 mM glucose, 80% and 60% lower levels respectively, than the Wistar islets (*N* = 3, p<0.001) (Fig. 1C).

### Global miRNA profiling of pancreatic islets of GK rats and Wistar controls

**Figure 2:**
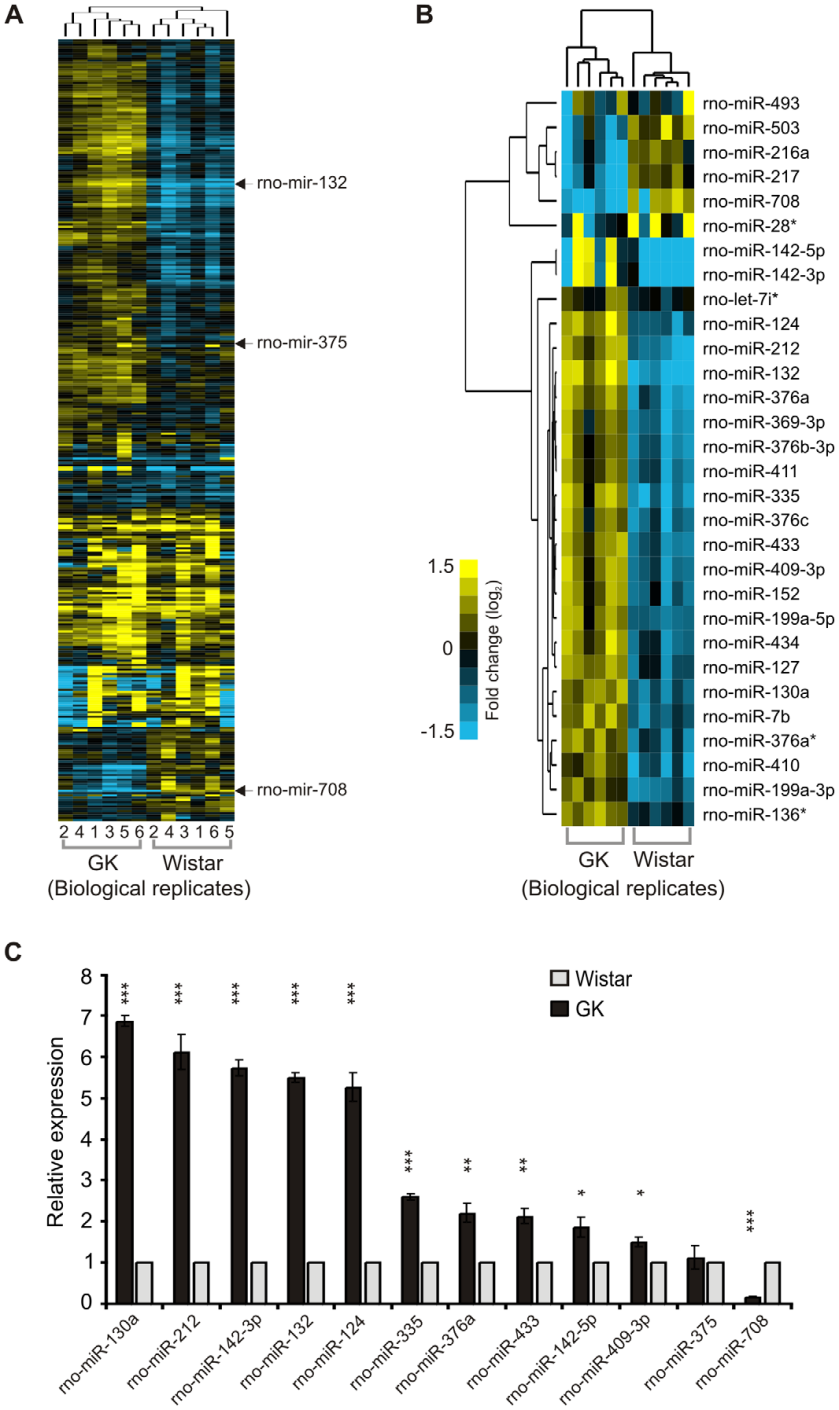
Global miRNA profiles of the pancreatic islets of GK and Wistar rats and qPCR validation. **A**. Hierarchical clustering of array signals from 348 rat miRNAs allowed for the separation of the individual animals into two groups, 6 GK *vs* 6 Wistar. Rat miR-375, miR-132 and miR-708 are indicated for reference, representing no significant change (dark tones), upregulated (yellow tones) and downregulated (blue tones) miRNAs in GK. **B**. Significant Analysis of Microarrays (SAM) identified 30 differentially-regulated miRNAs in the GK rat pancreatic islets, clustered into 6 downregulated and 24 upregulated miRNAs (median False Discovery Rate  =  0%). **C**. Stem-loop qPCR validation of selected 12 miRNAs in GK and Wistar islets. Mir-375 was included as a non-regulated control. Each miRNA was normalized to the geometric mean of U6 snRNA and U87 rat expressions as implemented in GeNorm v3.5. The 2^-ΔΔCt^ method was used for relative quantification using the Wistar expression level as calibrator. The presented data are the average of *N* = 3 biological replicates performed independently each in triplicate qPCR wells ± SEM; (*) *P*<0.05, (**) *P*<0.01, (***) *P*<0.001 GK *vs* Wistar.

Hierarchical clustering of the global miRNA profiles comprising 348 rat miRNAs allowed clear separation of the GK rats from the controls (Fig. 2A). Significant analysis of microarrays (SAM) identified 30 differentially-regulated miRNAs, 24 of which are predominantly upregulated in the GK rat islets (Fig. 2B) (median False Discovery Rate, FDR* = *0%). Employing SAM without large fold-change threshold requirement allowed for the identification of differentially-regulated miRNAs with small but consistent differences among the individual animals from each group.

Using miRNA-specific stem-loop qPCR assays [24] we validated the expression of ten most upregulated miRNAs from the significant list, and also prioritizing miRNAs which appeared in previous studies on pancreatic islets or insulin-secreting beta cell lines such as miR-124, miR-376a, miR-132 and miR-212. We also included the most consistently down-regulated miRNA, rno-miR-708, in the GK rat islets for validation. As a negative control we included rno-miR-375, being found not to be differentially-regulated in the arrays (Figure S1B). In agreement with the array results, we observed significant upregulation in the qPCR of the selected miRNAs in the GK rat islets compared to the controls (p<0.05; Fig. 2C).

### Glucose regulation of differentially-expressed miRNAs in Wistar and GK islets

Since one of the major diabetic phenotypes of GK rats is impaired GSIS, we investigated whether the differentially-expressed miRNAs would show glucose-dependent regulation both in the short-term (1 h) and long-term (24 h) exposure to varying glucose concentrations. The three glucose concentrations used were 2.8 mM (2.8G), 8.3 mM (8.3G) and 16.7 mM (16.7G), representing hypoglycaemic, near-physiological and hyperglycaemic environments.

**Figure 3:**
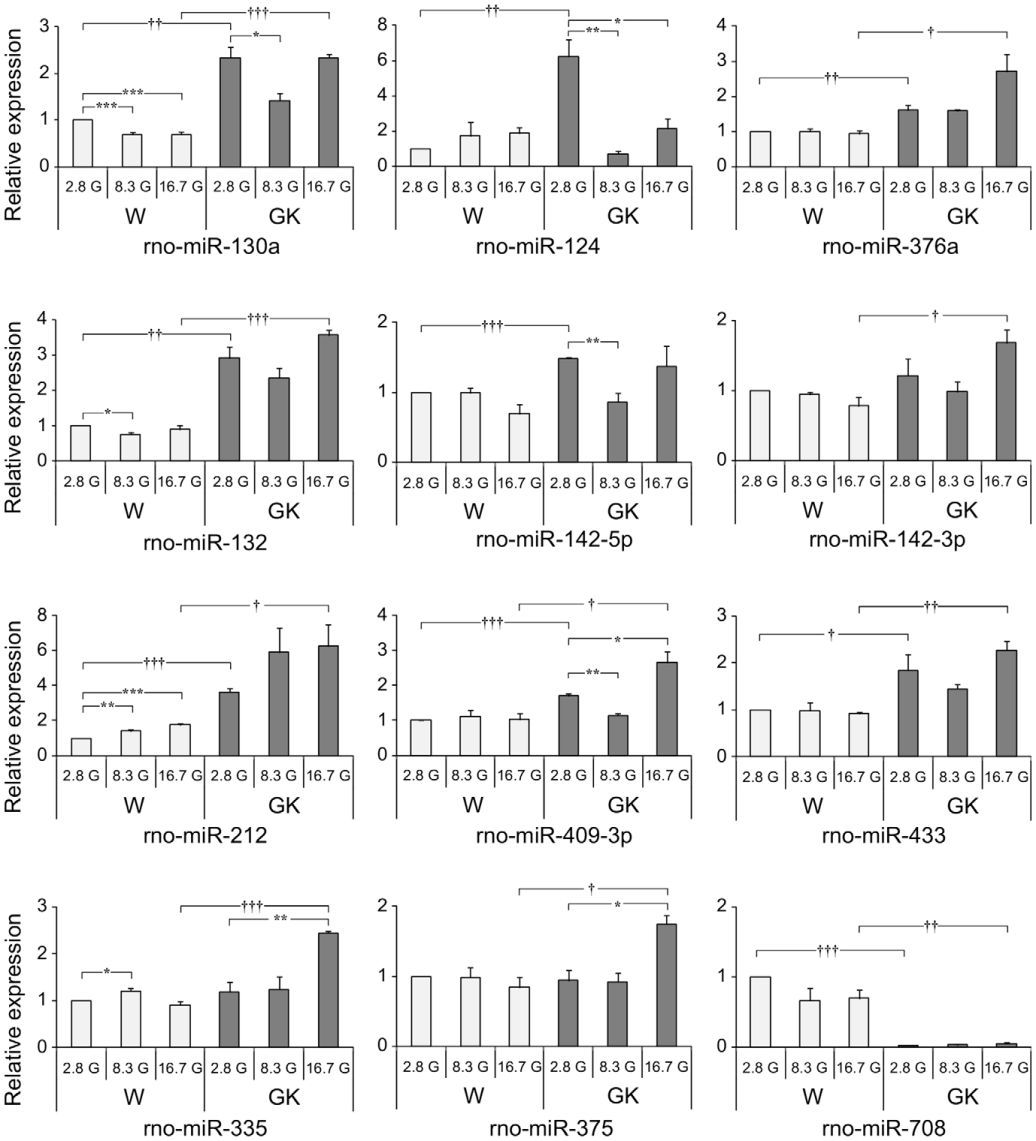
Glucose-dependence of miRNA expression after 1 h incubation at 2.8 mM, 8.3 mM and 16.7 mM glucose. More pronounced variation of miRNA expression trajectories (magnitude and direction of expression) were observed in the GK compared to Wistar islets across the different glucose concentrations. Each miRNA was normalized to the geometric mean of U6 snRNA and U87 rat. The 2^-ΔΔCt^ method was used for relative quantification using Wistar expression level at 2.8G as calibrator. The presented data are the average of *N* = 3 biological replicates performed independently each in triplicate qPCR wells ± SEM. Intra-sample (within same animal group) significance denoted by (*) *P*<0.05, (**) *P*<0.01, (***) *P*<0.001 *vs* 2.8G of same animal group. Inter-sample (W *vs* GK) significance denoted by (†) *P*<0.05, (††) *P*<0.01, (†††) *P*<0.001, compares expression levels from different animal groups of the same incubating glucose concentration. Different y-axis scaling was used for each miRNA to allow easy comparison of expression levels across different conditions.

In the 1 h incubation (Fig. 3), with the exception of rno-miR-142-3p and rno-miR-335, the relative expression levels of the miRNAs at 2.8G were similar to those of freshly isolated islets (Fig. 3; p<0.05 *vs* 2.8G in Wistar), *i.e.* GK miRNAs significantly-upregulated in the microarrays consistently exhibited higher levels of expression than the Wistar miRNAs, whereas rno-miR-708 which was found to be downregulated in GK was minimally expressed. Among the miRNAs tested only rno-miR-130a, rno-miR-132, rno-miR-212 and rno-miR-335 responded to glucose stimulation at either 8.3 mM or 16.7 mM in the healthy Wistar islets (Fig. 3; p<0.05).

In GK, more pronounced variation of miRNA expression trajectories (magnitude and direction of expression) were observed upon glucose stimulation at 8.3G, especially for rno-miR-124, rno-miR-142-5p and rno-miR-409-3p (Fig. 3; p<0.01 *vs* 2.8G in GK). In fact, a number of miRNAs are markedly decreased at 8.3G. In virtually all upregulated miRNAs in the GK, their levels at 16.7G are significantly greater than the levels found in Wistar. Even rno-miR-375 which was non-responsive at stimulatory conditions in Wistar significantly increased in GK at 16.7G (Fig. 3; p<0.05).

**Figure 4:**
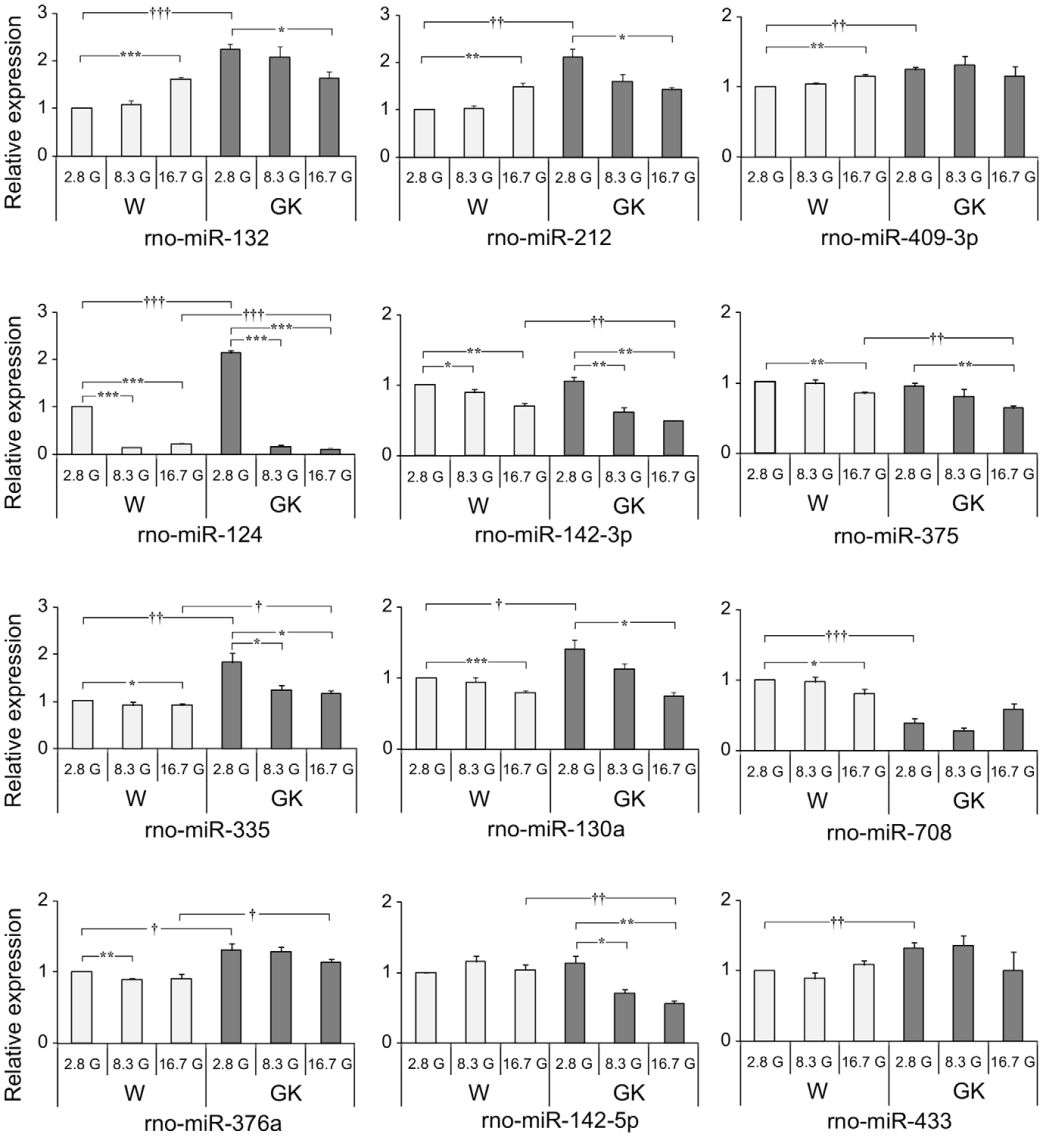
Glucose-dependence of miRNA levels after 24 h incubation at 2.8 mM, 8.3 mM and 16.7 mM glucose. Three general trends of miRNA expression trajectories were observed for Wistar islets at 2.8G *vs* 16.7G: i) increased expression as exhibited by rno-miR-132, rno-miR-212 and rno-miR-409-3p, ii) decreased expression as in the case of rno-miR-124, rno-miR-142-3p, rno-miR-375, rno-miR-335, rno-miR-130a and rno-miR-708 and, iii) no change as seen in rno-miR-376a, rno-miR-142-5p and rno-miR-433. For GK islet expression, the miRNAs generally exhibited expression trajectories aimed at attaining Wistar islet levels. Data analysis and presentation are as described for Figure 3.

For islets incubated 24 h in 2.8 mM glucose, the expression levels of the miRNAs in the GK also reflected the results found in the miRNA arrays of freshly isolated islets, except for rno-142-3p and rno-142-5p (Fig. 4). Nonetheless the latter two still showed a tendency to be at higher levels in the GK than in the Wistar. In general, aside from more significant changes in expression levels of miRNAs at 24 h incubation compared to 1 h incubation, three trends in terms of expression changes are also observed in the Wistar islet upon stimulation at 16.7G as compared to 2.8G: i) increasing miRNA levels, as displayed by rno-miR-132, rno-miR-212 and rno-miR-409-3p, ii) decreasing miRNA levels as exhibited by rno-miR-124, rno-miR-142-3p, rno-miR-375, rno-miR-335, rno-miR-130a and rno-miR-708, and iii) no significant change as displayed by rno-miR-376a, rno-miR-142-5p and rno-miR-433.

In comparison, the expression trajectories of some miRNAs in the GK islets also follow similar trends as in the Wistar islets, such as rno-miR-124, rno-miR-142-3p and rno-miR-375. In contrast, rno-miR-212 and rno-miR-132 showed the opposite trend leading to decreased miRNA levels upon stimulation at 16.7 G. Thus, for miRNAs that increases with increasing glucose concentrations in the normal Wistar islets, the GK islet miRNAs levels go down (rno-miR-132 and rno-miR-212) or do not change (rno-miR-409-3p), whereas for miRNAs whose levels decrease with increasing glucose concentration in Wistar islets, the GK islet miRNAs is also reduced (*e.g.* rno-miR-124, rno-miR-142-3p, rno-miR-375) (Fig. 4).

These results indicate compensatory mechanisms in the GK islets striving to reset the levels of miRNAs to that of the controls during prolonged exposure to hyperglycaemic condition. This is clearly seen for rno-miR-212, rno-miR-132, and rno-miR-130a, whose expression levels in the GK and Wistar islets eventually coincide at 16.7G (Fig. 4). However, despite these attempts in the GK islet to attain normal levels of miRNAs, failures were seen in most GK miRNAs wherein the levels at 16.7G either overshoot those of Wistar's as in rno-miR-142-3p, rno-miR-142-5p, rno-miR-375 and rno-miR-124, or completely miss the normal levels as in the case of rno-miR-335 and rno-miR-376a (Fig. 4).

### Gene ontology enrichment of the putative targets of co-regulated miRNAs

Next we were interested to find potential targets proteins of miRNAs up-regulated in the GK rat. The poor overlap between different miRNA target prediction algorithms is well known and the lack of rationale behind filtering of targets by using multiple target prediction algorithms has been demonstrated [25]. Since spatio-temporal co-expression of mRNA and miRNAs is more important in assessing reliability and accuracy of target predictions than conservation scores [26], we chose to exclusively use TargetScan Release 5.1 (April 2009) [27] to retrieve all putative targets, both highly-conserved and rat-specific miRNA targets. Target Scan predicted a total of 13591 target genes for the ten co-expressed miRNAs upregulated in the GK rat (data not shown).

**Figure 5:**
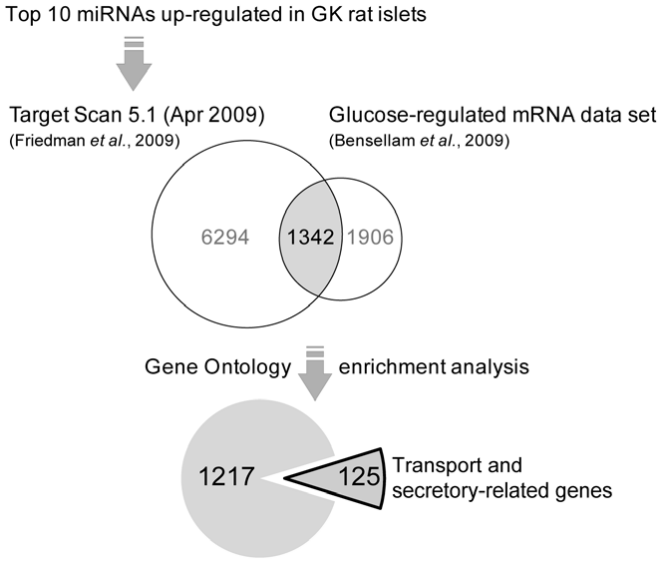
*In silico* strategy to analyze the miRNAs upregulated in the GK rat islets. One of the highlights of the approach is the filtering of predicted targets with known glucose-regulated mRNA data set from a previous study, significantly reducing false positive targets. This also resulted to a more focused enrichment of genes already implicated in islet functions.

To reduce the amount of false positive hits, the predicted targets were then filtered by intersecting with a list of glucose-regulated mRNAs expressed in isolated rat islets incubated in low, medium and high glucose levels [28] (Fig. 5). This resulted to 1342 glucose-regulated target genes in the rat pancreatic islets (Table S1). Gene ontology enrichment aiming for the biological process category revealed functional clusters significantly enriched for genes involved in transport and secretory processes (Table 1). The functional annotation clustering is based on the idea that similar gene members would be expected to fall into similar annotation groups. Indeed the annotations between the two clusters are highly related with a combined 125 genes among the different gene ontologies. Of these genes, ∼50% (60/125) have more than one miRNA predicted target sites (Table S2). Table 2 shows representative target genes known to be involved in insulin exocytosis potentially regulated by miRNAs.

**Table 1:** Gene ontology enrichment of the putative gene targets of ten upregulated GK islet miRNAs.

Functional Group 1				
Gene ontology (GO) term	Gene count	Fold Enrichment	P-value	Bonferroni corrected
GO:0015031∼protein transport	87	2.1	2.78E-11	6.86E-08
GO:0046907∼intracellular transport	96	2.0	8.04E-11	1.98E-07
GO:0006886∼intracellular protein transport	63	2.2	8.18E-09	2.02E-05

Functional Group 2				
Gene ontology (GO) term	Gene count	Fold Enrichment	P-value	Bonferroni corrected
GO:0045045∼secretory pathway	44	2.3	3.55E-07	8.76E-04
GO:0006888∼ER to Golgi vesicle-mediated transport	17	4.4	5.46E-07	1.35E-03
GO:0048193∼Golgi vesicle transport	22	3.2	3.49E-06	8.58E-03
GO:0032940∼secretion by cell	46	2.0	5.27E-06	1.29E-02

1342 glucose-regulated putative gene targets (listed in Table S1) of ten upregulated miRNAs in GK rat islets yielded two statistically-significant clustering of Gene Ontology (GO) terms characterising transport and secretory processes.

### Targeting of rat Stxbp1-3′UTR by rno-miR-335

**Figure 6:**
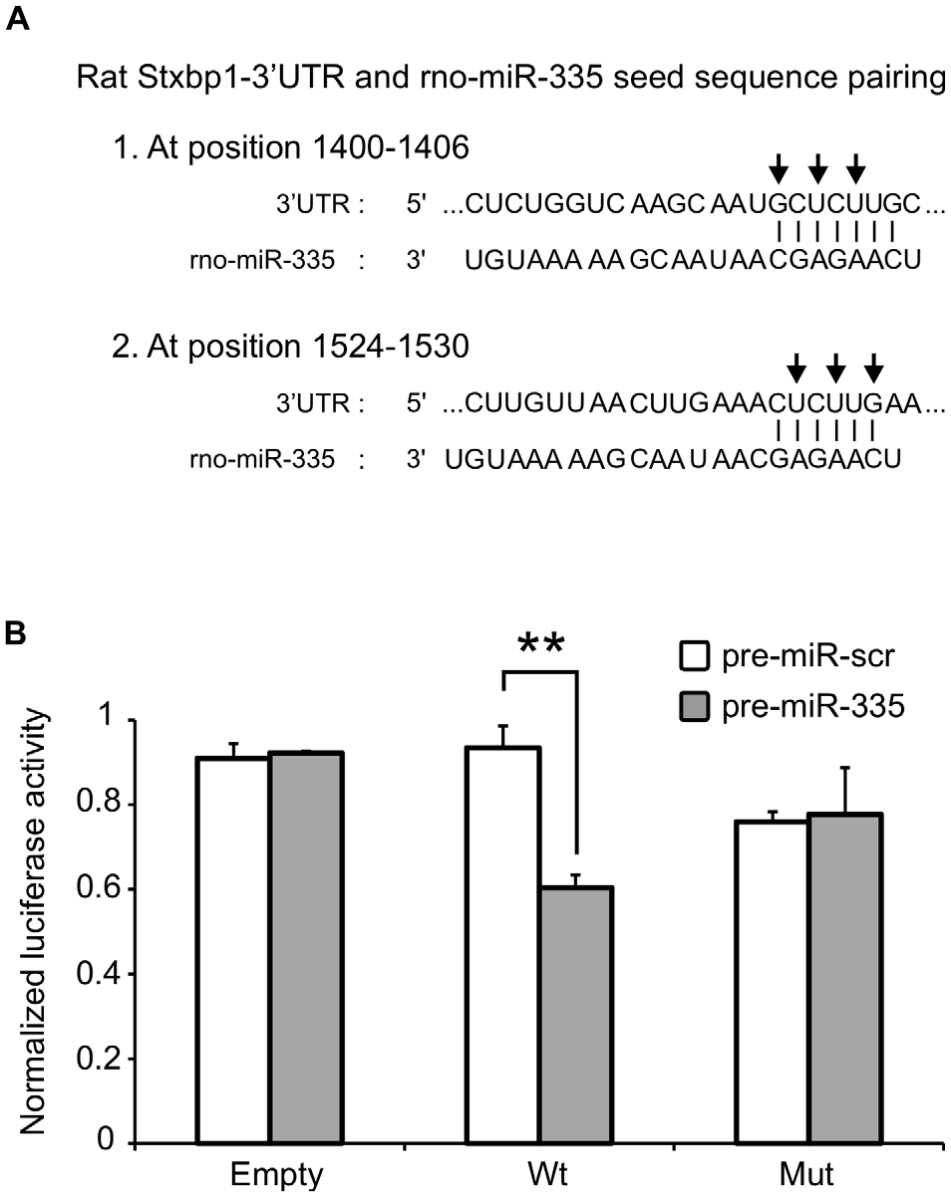
Interaction of rno-miR-335 with the predicted binding sites in the 3′UTR of rat Stxbp1. **A**. The rat Stxbp1 3′UTR contains two putative miR-335 binding sites as predicted by TargetScan 5.1. The two sites are located in the proximal region and both could be included in a 200 bp fragment cloned into a dual luciferase reporter plasmid. Arrows indicate the sites in the seed sequences that were mutated into complementary bases to act as negative control in the luciferase assay **B**. Two inserts, one with the wildtype rat Stxbp1-3′UTR sequence (Wt) and the other with sequences mutated at both miR-335 predicted binding sites (Mut) were cloned into the pmiRGLO dual luciferase vector. The pmiRGLO dual luciferase vector alone (Empty) was included as positive controls. HeLa cells were co-transfected with the empty vector or plasmid constructs and pre-miR-335 or pre-miR-scr (control with scrambled sequence) and assayed after 48 hours. Transfection efficiency was normalized using the *Renilla signal.* Data represents two independent transfections ± S.E.M. with n = 3. (**) *P*<0.01.

Stxbp1 (Syntaxin-binding protein 1) or Munc18-1 plays an important role in the fusion of insulin secretory granules in the plasma membrane of the beta cell by modulating the folded conformation of syntaxin 1A [29]. Not surprisingly, the expression level of Stxbp1 is reduced in the islets of both T2D patients [19] and GK rats [18]. The proximal region of the rat Stxbp1 3′UTR contains two miR-335 binding sites as predicted by TargetScan (Fig. 6A). Two dual luciferase reporter constructs, one with wildtype and another with mutant sequence in the miR-335 binding site seed regions were constructed. The inserts are ∼200 nt long encompassing the two miR-335 binding site of the Stxbp1 3′UTR. Co-transfection of pre-miR-335 with the reporter plasmid containing wildtype seed sequence into HeLa cells showed a ∼30% reduction in the luciferase signal (Fig. 6B) indicating a binding interaction between the miRNA and the target sites. This interaction is lost when the plasmid construct with mutations in the seed sequence of miR-335 binding site was used instead (Fig. 6B). These results were consistent and were also observed in the insulin-secreting cell line, INS1-832/13 (data not shown). Altogether, these results suggest the direct targeting of rat Stxbp1 by rno-miR-335.

## Discussion

We have shown differential miRNA expression profiles in the isolated pancreatic islets of mildly hyperglycaemic GK rats compared to that of healthy Wistar controls. One of the strengths of this work lies in the use of LNA-based arrays. In such arrays, the capture oligonucleotide probes were adjusted within a narrow melting temperature range by introducing LNA monomers yielding optimal hybridisation conditions. Recently, the utility and reliability of these arrays were shown to be comparable, if not superior to next generation high-throughput sequencing approaches to expression profiling [30]. Thus, in combination with profiling of as many as six individual biological replicates from each phenotypic group, our array results validated by qPCR experiments undoubtedly identified genuine miRNA expression differences in the pancreatic islets of the two sets of animals.

Four of the glucose-regulated miRNAs found in this study, mir-124, mir-212, mir-132, and mir-409-3p, were previously reported to be upregulated in the mouse insulin secreting cell line, MIN6, after 16 h stimulation in 25 mM glucose [31], which is noteworthy despite that tumour-derived cell lines have been shown to generally exhibit significantly different miRNA signature compared to primary cells [6].

Interestingly, miR-212 and miR-132 have also been shown to be upregulated in isolated pancreatic islets in the obese phenotypes of both the diabetes-resistant (B6) and diabetes-susceptible (BTBR) mouse models [32]. The deregulated expression of mir-132 and mir-212 in different experimental models strongly links these miRNAs to common pathways underlying the disease pathologies of diabetic GK rats and obese mouse models.

We found that incubation of isolated rat islets in different glucose concentrations and lengths of time revealed differences in both magnitude and direction of miRNA expression between the model animals and the controls. We saw changes in miRNA expression manifested within a short temporal window of only one hour, such as for rno-miR-130a, rno-miR-132, rno-miR-212 and rno-miR-335. These miRNAs are most likely regulated by hyperglycaemia rather than the GK phenotype *per se* since they responded to glucose stimulation even in the Wistar islets.

A further evidence of active regulation of miRNAs during short-term fluctuation of glucose levels is the prominent decrease of some miRNAs at 8.3G, which is a clear deviation from the typical up or down glucose concentration-dependent mode of regulation. Interestingly, this was also reported in the work of Bensellam and co-workers in which more than 400 mRNA probe-sets displayed atypical expression patterns, *e.g.* V- or inverse-V- shaped across four glucose concentrations. Furthermore, statistically-significant changes in gene expression were predominantly observed between G5 and G10 indicating active gene regulation mechanisms aimed at keeping optimal islet function during physiological glucose levels [28]. In our 1 h incubation time, we also observed more significant changes in miRNA expression from 2.8G to 8.3G in both the Wistar and GK islets. However, the phenomenon of markedly decreased miRNA levels at 8.3 G was more seen in the GK islets than in the controls indicating greater requirement in the diseased animals to lower the amount of these negative regulatory molecules.

Rapid modulation of cellular miRNAs was previously observed in human hepatoma cell line and primary murine hepatocytes where IFNβ treatment lead to induction of specific miRNAs within just 30 minutes of incubation [33]. In another study the presence of discrete populations of miRNAs in specific regions of the human brain with half-lives in the order of minutes-to-hours suggested the presence of a rapidly deployed miRNA-dependent signalling system in central nervous system gene expression [34]. In the case of pancreatic islet cells, we speculate that the glucose-regulated miRNAs could be envisioned as vital components of a rapid physiological response mechanism activated during nutrient-induced insulin secretion. Indeed, among the filtered predicted targets of the upregulated miRNAs in the GK rats in particular rno-miR-335, are enriched for several key exocytotic proteins (Table 2), some of which were shown to have reduced expression in human T2D cases including Stxbp1 (Munc18-1) whose 3′UTR fragment we have shown to interact with miR-335.

**Table 2:** Representative target genes from the enriched GO categories known to be involved in insulin exocytosis.

Entrez ID	Gene Name	Gene Symbol	Comment	miRNA
25012	Synaptosomal-associated protein 25	SNAP25	t-SNARE protein	rno-miR-409-3p, rno-miR-335
64630	Synaptosomal-associated protein 23	SNAP23	t-SNARE protein	rno-miR-124
81802	Syntaxin 3	STX3	t-SNARE protein	rno-miR-142-5p, rno-miR-132
80843	Sec 61, alpha subunit (S. cerevisiae)	SEC61A1	role in the insertion of secretory and membrane polypeptides into ER	rno-miR-130a, rno-miR-212
25558	Syntaxin binding protein 1	STXBP1	Munc18-1; SNARE accesory factor	rno-miR-433, rno-miR-335
60355	N-Ethylmaleimide sensitive fusion protein	NSF	involved in the disruption of the SNARE-complex after fusion	rno-miR-142-3p, rno-miR-132, rno-miR-376a
140594	Synaptotagmin-like 4	SYTL4	granuphilin/Slp-4; important in docking of insulin-containing granules	rno-miR-132
116657	Complexin-2	CPLX2	possible minor role in insulin exocytosis; 80% similar to CPLX1 involved in GSIS	rno-miR-335
56768	Piccolo	PCLO	complexes with Epac2; involved in cAMP-dependent exocytosis	rno-miR-142-5p, rno-miR-433, rno-miR-142-3p
64088	Sorting nexin 16	SNX16	effects on EGF receptor trafficking	rno-miR-142-5p, rno-miR-132, rno-miR-142-3p, rno-miR-409-3p

Table S2 contains the full list of enriched target genes and putative regulating miRNAs.

Islets incubated for 24 h at 2.8G and 16.7G represent exposure to chronic states of hypo- and hyperglycaemia, respectively. Such extended exposure of islets to non-physiological glucose environments is more aimed at studying the effects of nutrient availability and in the case of higher glucose concentrations, also imposes effects of gluco-toxicity on islet function. A longer incubation time also allows for the observation of constitutively regulated miRNAs. Notably, during this period we found evidence of dosage compensatory mechanisms attempting to bring the expression levels of specific miRNAs within the islets of the GK animals towards that of the controls. This trend was particularly distinct for both miR-212 and miR-132, which belong to the same gene cluster being only 200 nt apart and containing identical seed sequences. Additionally, the genes encoding both miRNAs harbour a cAMP-response element binding protein (CREB) site indicating common regulation by the same transcription factor [35]. The observed deregulation of these miRNAs in the GK islets during GSIS may not necessarily be a negative consequence of the disease phenotype but could also be a way to reset the relevant proteins to wild-type levels.

The presence of deregulated miRNAs in the GK rat islet is strikingly clear, but how do miRNAs ultimately contribute to the islet's specialized function as a hormone-secreting factory? In a recent review, D.P. Bartel (2009) outlined different scenarios of miRNA-mediated regulatory effects, wherein miRNAs may act as binary off switches, rheostats or as facilitators of neutral interactions [26]. The pancreatic islets are source of hormones with opposing effects and are subjected to constantly fluctuating signals. It is rather intuitive that such a complex system would require fine tuning mechanisms to ensure optimal levels of regulatory proteins at any given time. Thus, miRNA as rheostat capable of regulating gene expression for optimal protein output [36] would be a likely component of such fine tuning interaction for the proper functioning of pancreatic islets. Disruption of this balancing mechanism ultimately leads to impaired cellular processes triggering abnormal physiological manifestations as seen in many human diseases.

It is well-known that the lower capacity of GK islets to secrete insulin under glucose stimulatory conditions is not due to reduced stores of islet hormones since insulin content in GK islets and controls are comparable [22]. These results are compatible with the idea that one of the primary causes of the diabetic phenotype in GK rats lies in a defective exocytotic machinery in the islets [16]. In fact careful electrophysiological measurements on GK rat beta cells strongly indicate that the events downstream of glucose metabolism in the canonical GSIS mechanism are defective [37]. As in the case of type 2 diabetic humans, the GK rat diabetic phenotype is also characterized by downregulation of key exocytotic proteins in the pancreatic beta cell [15]. Specifically, deficiencies in the SNARE complex proteins have been shown to impair insulin secretion [17]. Altogether, previous knowledge about the GK phenotype supports our computationally-predicted list of target genes enriched for genes involved in transport and secretion processes, containing genes known to play central roles in insulin exocytosis (Table 2). This is further strengthened by our experimental validation of rno-miR-335 interaction with Stxbp1 3′UTR (Fig. 6).

To conclude, we hypothesize that the imbalance in the miRNA network in the GK rat islets may contribute to the impaired GSIS characteristic of the animals, though it cannot be excluded that such perturbation may serve as a resetting/compensatory mechanism to counteract the disease phenotype. It should also be noted that the *in vitro* experiments performed on isolated pancreatic islets may only partially reflect the dynamic nature of miRNA regulation happening *in vivo.* Nonetheless, the potential role of miRNAs in regulating the insulin exocytotic machinery will pave further investigation about the contribution of deregulated miRNAs in T2D. Translating these findings into the context of human T2D and further characterization of other hypothesized miRNA-target interactions would certainly contribute to understanding the roles of these relatively novel regulatory molecules in the aetiology of the disease.

## Materials and Methods

### Ethics Statement

All animal experimentations were approved by the local ethics committee of Lund University under permit number M 185 06 and M 73 08.

### Experimental animals, monitoring of glycaemia and plasma insulin

Female GK/MolTac and control Wistar rats, 60 days old, were purchased from Taconic Europe. The GK inbred model was developed at Tohoku University, Japan in 1975 [14]. GK/MolTac rats develop T2D at around 14–16 weeks of age (http://www.taconic.com). Non-fasting glucose levels were measured from intra-cardiac blood using a glucose meter (ACCU-CHEK Aviva, Roche) immediately after killing. Plasma insulin was measured by RIA. Due to insufficient amount of islets from individual animals, separate batches of animals were used for in vitro insulin-secretion assay and global profiling of miRNA expression.

### Islet preparation and in vitro insulin-secretion assay

Pancreatic islets were isolated by collagenase digestion and the islets were handpicked in cold Hank's buffer with 1 mg/mL bovine serum albumin (BSA) prior to secretion assays. Islets from three individual animals (no pooling) were seeded in separate wells in plates containing Krebs buffer with 2.8 mM glucose. After 1 h pre-incubation at 37°C with 5% CO_2_, the islets were transferred in new plates with fresh medium at desired glucose concentrations and incubation time. In parallel, batches of 10–12 intact islets were transferred into new tubes in quadruplicates for 1 h or 24 h GSIS. Samples were retrieved from the tubes for insulin secretion assays using RIA. The islets from plates were collected and processed immediately for total RNA preparation and stem loop qPCR.

### Global LNA-based miRNA expression profiling and data analysis

Total RNA from freshly isolated islets from individual animals was extracted using the Qiagen miRNeasy isolation kit (Qiagen, Hilden, Germany). The quality of the total RNA was evaluated both by spectrophotometry and electropherogram profiles using Nanodrop and Experion's automated electrophoresis system (Bio-Rad). All samples have Abs 260/280 ∼2.0, Abs 260/230 >1.4 and Experion's RNA Quality Indicator (RQI) >7.0, indicating high-quality total RNA preparations and consistent extraction procedure (data not shown). A reference pool was made from mixing equal amount of total RNA from the individual animals. 500 ng of total RNA from each sample and reference pool were labelled with miRCURY™ Hy3™ and Hy5™ fluorescent label (Exiqon, Denmark). The Hy3™-labeled samples and a Hy5™-labeled reference pool RNA samples were mixed pair-wise and hybridized to the miRCURY™ LNA array v.11.0 (Exiqon, Denmark) according to manufacturer's protocol for Maui hybridisation chamber. The LNA array contains capture probes targeting 348 rat miRNAs listed in the miRBASE version 12.0 release [38]. Images were acquired using an Agilent array scanner and spot signals were processed (default background subtraction) and quantified using GenePix Pro 4.1. Intensities within arrays were normalized using the global Lowess (LOcally WEighted Scatterplot Smoothing) regression algorithm as implemented in the Comprehensive R-based Microarray Analysis web front end (CARMAweb 1.4) [39]. The log2 values of the difference between the sample and reference signals were used for hierarchical clustering using Cluster 3.0 [40] and the values were visualized with Java TreeView 1.0.12 [41].

### miRNA-specific stem-loop qPCR

Total RNA from all islet samples were prepared and quality checked as above. MiRNA expression was measured by stem-loop qPCR using primers and probes from TaqMan MicroRNA assays (Applied Biosystems). For each experimental condition, three separate real-time PCR runs were performed from islet preparations from different individuals (*N = *3), each in triplicate wells in 384-well plates using Applied Biosystems 7900HT RT-PCR system.

U6 snRNA and U87 rat showed stable expression levels in all conditions tested in the two groups of animals (Figure S2) and both were used as endogenous controls. The method described in GeNorm [42] was used to derive normalization factors from the multiple internal control genes and relative quantification was performed using the 2^−ΔΔCt^ method. Wistar expression levels were used as calibrators in qPCR on fresh islets, while Wistar expression level at 2.8 mM glucose were used as calibrators in qPCR on GSIS experiments.

### In-silico prediction and functional analysis of miRNA targets

TargetScan Release 5.1 (April 2009) [27] was used to retrieve all putative targets, both highly-conserved and rat-specific miRNA targets. The predicted targets were then intersected with a list of glucose-regulated mRNAs expressed in rat islets, supplementary material of Bensellam et al (2009) [28], which were re-annotated with the latest available Affymetrix annotation file, Rat230_2.na30.annot.csv (Release 30, 11/15/2009). Gene ontology enrichment on the final gene list (Table S1) was performed using DAVID 2008 tools as described [43]. Figure 5 summarizes the analysis strategy.

### Plasmid construction and dual luciferase assay

To generate the inserts encompassing the two rno-miR-335 binding sites in the proximal region of Stxbp1-3′UTR, single-stranded oligonucleotide sequences (ultramers^TM^) were purchased (Integrated DNA Technologies, Inc. Germany). The sequences included position 1368 to 1547 (TargetScan numbering) of the 3′UTR of Stxbp1 (NM_003165). To facilitate directional insertion into the pmiRGLO dual luciferase miRNA target expression vector (Promega, USA; Cat#E1330), *PmeI* and *NotI* restriction sites were also added in the 5′end and *XbaI* site in the 3′end (underlined italics). The sequences of the 195 nt strands are: i) sense strand: 5′-*AAAC*TA*GCGGCCGC*CTTCTTTTGTCCCTTCTCTCTGGTCAAGCAAT**GCTCTTG**CTTCAGGACCTTGTTTGTCGAACATGTGGGGTTTCCTTTATGTTATTTATATAAATAATTTCTCAAATGGATATTTAAAAAAAAAGCTAGTCTGTCTTGAAACTTGTTAACTTGAAA**CTCTTGA**ATCTCAGTGTTTAAAGT*T* and ii) antisense strand: 5′-*CTAGA*ACTTTAAACACTGAGAT**TCAAGAG**TTTCAAGTTAACAAGTTTCAAGACAGACTAGCTTTTTTTTTAAATATCCATTTGAGAAATTATTTATATAAATAACATAAAGGAAACCCCACATGTTCGACAAACAAGGTCCTGAAG**CAAGAGC**ATTGCTTGACCAGAGAGAAGGGACAAAAGAAG*GCGGCCGC*TA*GTTT* . These strands were allowed to form a duplex prior to ligation within the multiple cloning site downstream of the *luc2* reporter gene in the linearized vector. This resulted to the dual-luciferase reporter containing wildtype rno-miR-335 binding seed sequences (bold sequences). Another pair of ultramers containing mutations (bold-underlined mutated into complementary bases) in the two rno-miR-335 seed sequence binding sites is likewise inserted into a separate pmiRGLO vector resulting to the mutant construct. Sequences and orientation of inserts in both constructs were verified by sequencing (GATC Biotech, Germany).

A day before transfection, ∼6000 HeLa cells were seeded in 96-well plate containing MEM (Invitrogen; Cat#10370) supplemented with 2 mM L-glutamine, 1 mM sodium pyruvate, 10% heat inactivated fetal bovine serum (Sigma #7524), 100 U/ml penicillin and 100 µg/ml streptomycin (Sigma # P0781) while ∼30000 INS1-832/13 cells were seeded in 96-well plate containing RPMI 1640 (Gibco Cat #21875) with 11.1 mM D-glucose supplemented with 10% heat inactivated fetal bovine serum (Sigma #7524), 100 U/ml penicillin and 100 µg/ml streptomycin (Sigma # P0781), 10 mM HEPES (Sigma #H0887), 2 mM L-glutamine (Sigma #G7513), 1 mM sodium pyruvate (Sigma #S8636) and 50 µM ß-mercaptoethanol, cultured at 37°C and 5% CO2 in a humidified atmosphere. The empty vector and plasmid constructs were then co-transfected with either pre-miR-335 or pre-miR-scrambled negative control #1 (Applied Biosystems Europe BV) to 60–70% confluent HeLa or INS1-832/13 cells using the Attractene transfection reagent (Qiagen GmBh, Germany; Cat.# 1051561). Transfection experiments were done independently for both cell lines two times in triplicate wells.

The Firefly luciferase activity was measured 2 days post-transfection using the Dual-Glo Luciferase assay system (Promega, USA; Cat.#E2920). Both cell lines exhibited ∼90% confluence by the time of assay. Transfection efficiency was normalized using the *Renilla* signal. Luminescence was acquired using the Magellan software on Tecan Infinite M200 plate reader.

### Statistical analysis

Insulin secretion, luciferase assays and qPCR data are presented as mean ± SEM and p values are from two-tailed *Student's* t test. Values of p<0.05 were considered as statistically significant. For LNA arrays, Significant Analysis of Microarrays (SAM) was performed as implemented in MeV v4.3 for two class unpaired without using the fold-change criterion [44]. Briefly, SAM two class unpaired is similar to a between subjects t tests wherein mean expressions in two groups are adjudged significant based on the calculated statistic, *d*, from the original and permuted data for each gene. The observed *vs* expected *d* values may be adjusted using a *Delta* parameter allowing for identification of significant genes even with small but consistent differences. SAM also estimates the False Discovery Rate (FDR) giving the proportion of significant genes most likely identified by chance. In this study, we set the *Delta* parameter to a conservative level corresponding to median FDR = 0%, *i.e.* the median number of false positives calculated during the procedure is equal to zero.

## Supporting Information

Figure S1 **Relative abundance of selected miRNAs in the pancreatic rat islet.** **A**. Quantile plots of miRNA signals from LNA (locked nucleic acid) arrays of total RNA of Wistar and GK pancreatic islets. The Hy3 fluorescence (sample signals) of miRNAs from six arrays of independent biological replicates from each animal group were averaged. **B**. Significant Analysis of Microarray (SAM) was performed on Hy3/Hy5 ratios (sample signal to reference signal ratio). Significant hits identified by SAM have fold-changes of at least 1.5.
(PDF)

Figure S2 **Evaluation of stability of endogenous controls for qPCR and array signals of the selected miRNAs.** **A**. Distribution of the average raw Ct values of each miRNA in the 14 qPCR conditions. **B**. Quantile plots of the average raw Ct values showing median expression levels and scatter of data points.
(PDF)

Table S1 List of 1342 rat genes which are intersection of glucose-responsive genes (Bensellam et al 2009) and collective miRNA targets in this study.
(XLS)

Table S2 List of 125 rat genes which are enriched for Transport and Secretory Processes with their corresponding putative miRNA regulator.
(XLS)
