# Supplementary figures and images for: Differential Glucose-Regulation of MicroRNAs in Pancreatic Islets of Non-Obese Type 2 Diabetes Model Goto-Kakizaki Rat
Source: PLoS One. 2011 Apr 7;6(4):e18613. doi: 10.1371/journal.pone.0018613 (PMC3072418; doi:10.1371/journal.pone.0018613)

**A**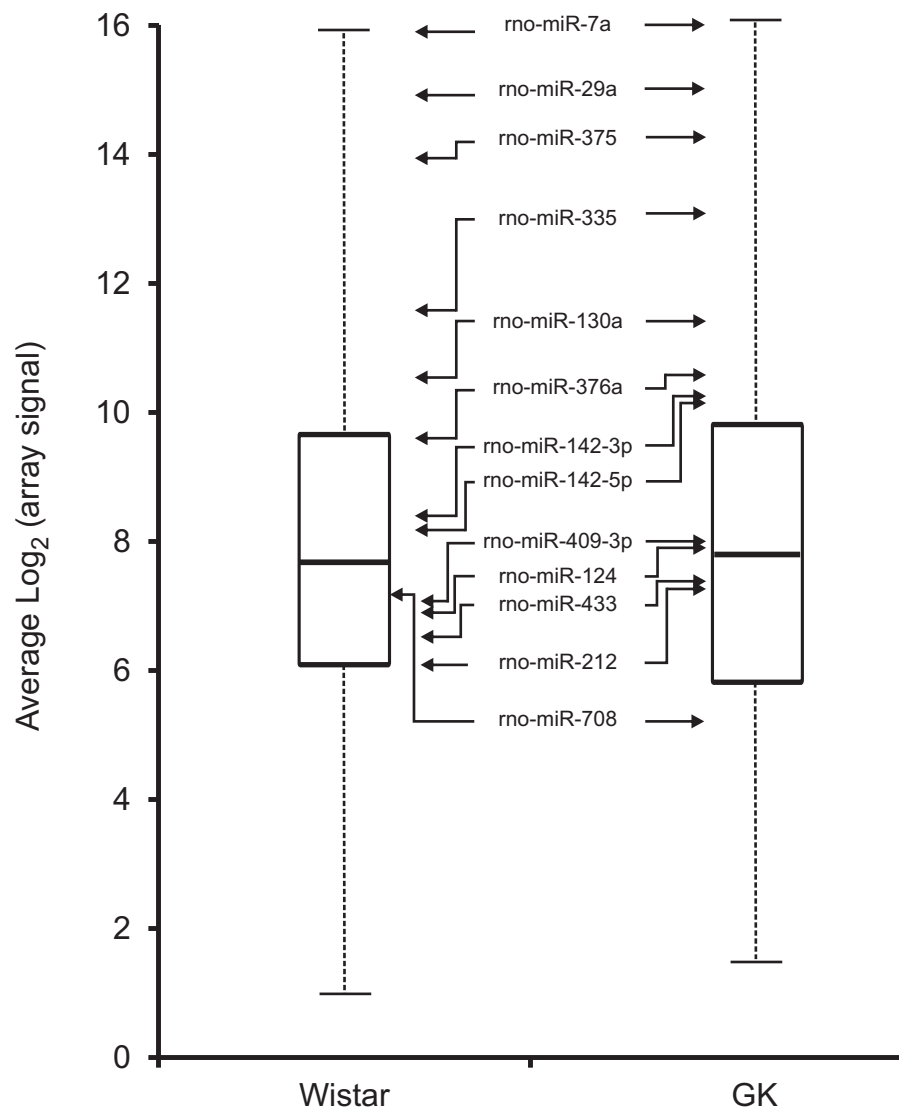**B**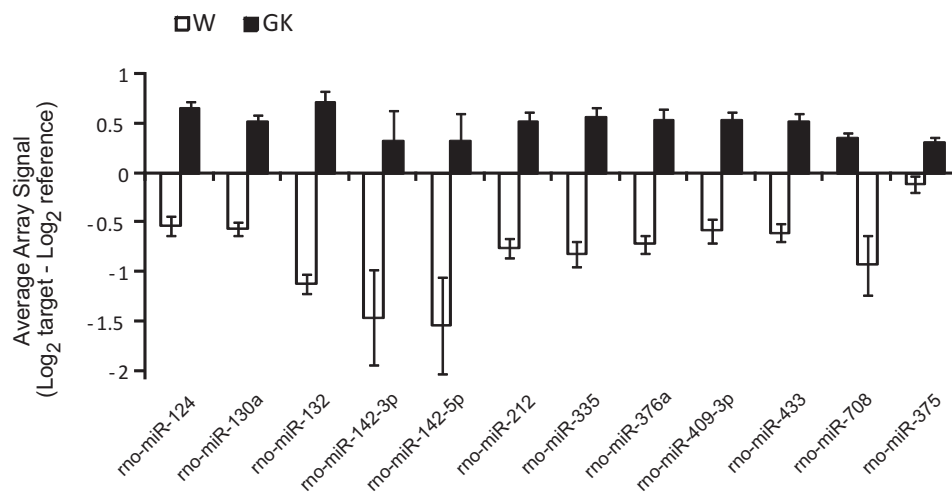

Supplement: Figure S1 — Relative abundance of selected miRNAs in the pancreatic rat islet. A. Quantile plots of miRNA signals from LNA (locked nucleic acid) arrays of total RNA of Wistar and GK pancreatic islets. The Hy3 fluorescence (sample signals) of miRNAs from six arrays of independent biological replicates from each animal group were averaged. B. Significant Analysis of Microarray (SAM) was performed on Hy3/Hy5 ratios (sample signal to reference signal ratio). Significant hits identified by SAM have fold-changes of at least 1.5. (PDF) [file pone.0018613.s001.pdf]

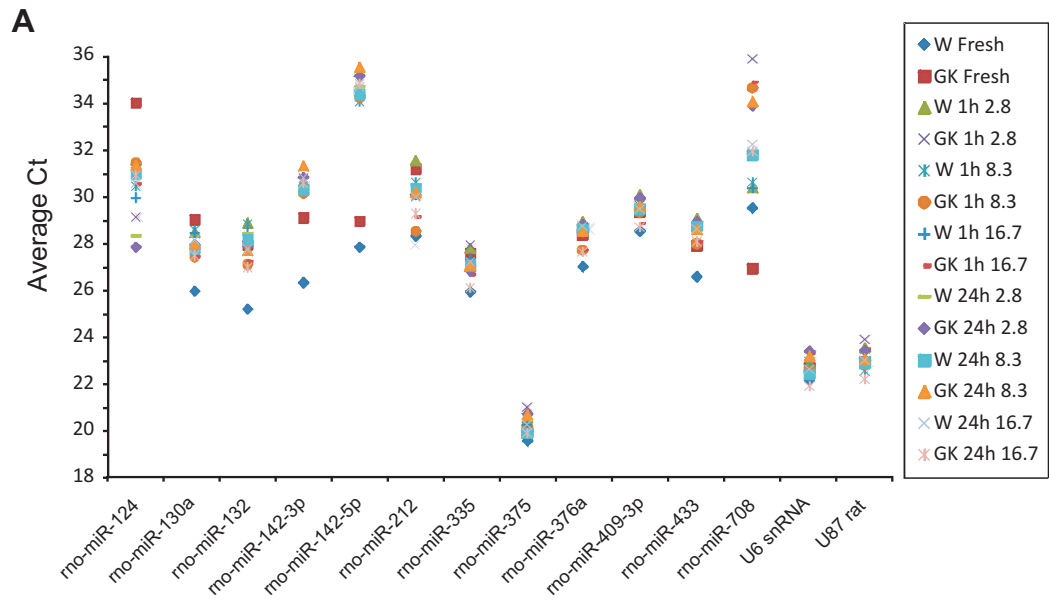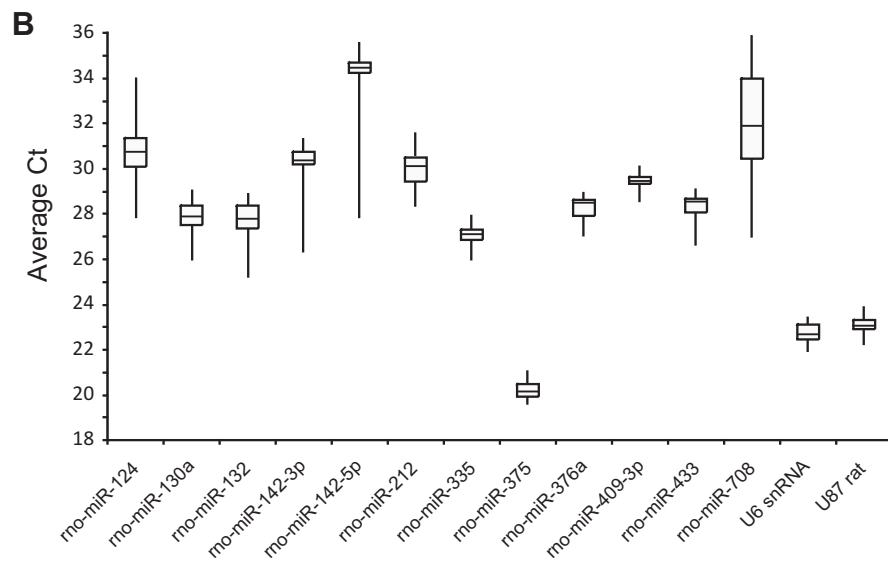

Supplement: Figure S2 — Evaluation of stability of endogenous controls for qPCR and array signals of the selected miRNAs. A. Distribution of the average raw Ct values of each miRNA in the 14 qPCR conditions. B. Quantile plots of the average raw Ct values showing median expression levels and scatter of data points. (PDF) [file pone.0018613.s002.pdf]
